# Awareness Among the Patients Under General Anesthesia: A Cross-Sectional Study

**DOI:** 10.7759/cureus.33567

**Published:** 2023-01-09

**Authors:** Dhana Lakshmi M, Ravi Madhusudhana, Suresh Kumar Naggaih

**Affiliations:** 1 Anaesthesia, Sri Devaraj Urs Medical College, Kolar, IND

**Keywords:** risk factors, dream, awareness, general anesthesia, brice's questionnaire

## Abstract

Introduction

Unless specifically asked, many patients may be hesitant to discuss their experiences. Some people might not recall what happened right after surgery, but they might remember it 1-2 weeks later. We undertook the current study to estimate the incidence of awareness among patients under general anesthesia (GA).

Methodology

We conducted a cross-sectional, analytical study for three months. The study included patients who underwent functional endoscopic sinus surgery (FESS), septoplasty, mastoidectomy, or laparoscopic appendicectomy under general anesthesia. Patients who refused to take part and had low Glasgow Coma Scale (GCS) scores (less than 9) or didn’t meet extubation criteria were all excluded from the study. We used a pre-validated semi-structured questionnaire for data collection. It had two sections. The first one includes demographic details, and the second section contains the modified Brice questionnaire. By using this questionnaire, we classified the patients as A, B, and C. Class A experiences are those that were remembered under anesthesia or surgery and were confirmed or disproved by the attending medical personnel present in the operating room. Class B, which stands for “potential awareness,” was defined as a state in which the patient could not specifically recollect any occurrence that occurred during anesthesia or surgery but could have made connections between memories and the surgical procedure. We define Class C as a lack of recalled intraoperative events with probable memories of scenarios from the immediate pre- or postoperative period. We analyzed the data collected using IBM Corp. Released 2012. IBM SPSS Statistics for Windows, Version 21.0. Armonk, NY: IBM Corp.

Results

About 240 patients took part in this study. Most of the people (68%) were men in the age group of 31 to 50 years. About 2% of the patient’s experience awareness during general anesthesia. Only 2.5% of patients experienced dreaming. The association between awareness and comorbidity was statistically significant (P < 0.001).

Conclusion

It is about to know that our study results suggest that awareness had an association with comorbidity among the patients undergoing surgery under general anesthesia.

## Introduction

One or two incidents of awareness under anesthesia may happen out of every 1000 patients receiving a general anesthetic (0.1%-0.2%) [1,2]. Incidence rates for obstetric and cardiac patients are higher overall, at 0.4 percent and 1.1-1.5 percent, respectively [3].

Unless specifically asked, many patients could be hesitant to discuss their experiences. Some people might not recall what happened right after surgery, but they might remember it 1-2 weeks later [4]. Therefore, a proper postoperative interview is the best technique for ascertaining intraoperative awareness. Most patients report having a faint auditory memory or a dream-like sensation, and this experience may not upset them too much. The patient may find their dreams to be quite unpleasant because they typically remembered more often than actual events [5].

About 36 percent of the patients reported feeling some kind of pain, from throat irritation to pain at the incision site. Although most awareness incidents are mild, some people have long-lasting, unfavorable effects like post-traumatic stress disorder and depression. There have been reports of late symptoms like anxiety, nightmares, and flashbacks in up to 33% of those who have experienced awareness [6].

With this above background, we did the current study to estimate the incidence of awareness among patients under General Anesthesia (GA).

## Materials and methods

Study design

We conducted a cross-sectional, analytical study in a tertiary care hospital in Kolar, Karnataka, India.

Study period

The study had been conducted for three months (1^st^ August to 31^st^ October 2022).

Ethical clearance

We did this study after getting ethical approval from the institutional ethics committee (DMC/KLR/IEC/513/2022-23).

Eligibility criteria and sampling

The study included patients who underwent functional endoscopic sinus surgery (FESS), septoplasty, mastoidectomy, or laparoscopic appendicectomy under general anesthesia. Patients who refused to take part and had low Glasgow Coma Scale (GCS) scores or didn’t meet extubation criteria were all eliminated from the study. Patients with a Glasgow Coma Scale (GCS) score of less than nine were excluded from the study, whereas those with a GCS score of 10 to 15/15 were included. The extubation criteria had the following conditions:

1) Adequate oxygenation, 2) adequate ventilation, 3) hemodynamically stable, 4) full reversal of muscle relaxation, and 5) neurologically intact.

We considered all the patients who fulfilled the eligibility criteria during the study period study participants (convenience sampling).

Data tool

We used a pre-validated semi-structured questionnaire for data collection. It had two sections. The first one includes demographic details such as age, sex, type of surgery, American Society of Anesthesiologists Classification (ASA) physical status, comorbid conditions (such as diabetes and hypertension), history of previous surgeries, and chronic drug intake or substance abuse. The second section contains the modified form of the Brice questionnaire, which is a standard questionnaire to elicit awareness among patients under general anesthesia [7].

Data collection

Following informed consent, we collected demographic data from study participants via a direct interview with patients. We kept a record of the patient's vital signs during the procedure and for the duration of the recovery period, including heart rate, oxygen saturation, electrocardiography, and non-invasive blood pressure. Before surgery, the patients were counseled by the investigator, and their informed permission was obtained. The type of anesthesia given was balanced anesthesia with a muscle relaxant. To determine the degree of neuromuscular blockage for the top-up of relaxants, we used Train of Four (TOF) monitoring.

After the surgery was over, we reversed the anesthesia, extubated the patient, and then moved her to the postoperative ward once she had successfully regained consciousness. We assessed the study participants about their intraoperative awareness using a questionnaire about an hour after they arrived from the post-operative ward.

By using this questionnaire, we classified the patients as A, B, and C. Class A experiences are those that were remembered under anesthesia or surgery and were confirmed or disproved by the attending medical personnel present in the operating room. Class B, which stands for “potential awareness,” was defined as a state in which the patient could not specifically recollect any occurrence that occurred during anesthesia or surgery but could have made connections between memories and the surgical procedure. We define class C as a lack of recalled intraoperative events with probable memories of scenarios from the immediate pre- or postoperative period.

A common instance of the unconscious state is sleep. Dreams can be remembered by people who have been awakened from various sleep stages. Although there was no dreaming while under anesthesia, some patients reported having dreams after waking up. It has been demonstrated that dream reports are still attainable even following properly executed and sufficiently deep anesthesia.

Statistical analysis

The data collected were entered into a Microsoft Excel sheet (Redmond, USA), and we analyzed the results using IBM Corp. Released 2012. IBM SPSS Statistics for Windows, Version 21.0. Armonk, NY: IBM Corp. Since almost all of the variables were qualitative, frequency and percentage were used to express them. And the Chi-square test was employed to determine the relationship between the aforementioned variables (if more than 20 percent of the cell value has an expected value less than 5, then Fischer exact test was used).

## Results

In total, about 240 patients took part in this study. Table 1 describes the baseline characteristics of the study participants. More than half (68 percent) of the study participants belonged to the age group of 31 to 50 years. Most of the participants were males (65 percent). We categorized over 3/4 of them as American Society of Anesthesiologists Classification (ASA) class II. Among them, nearly 21 percent of patients had comorbidities.

**Table 1:** Baseline characteristics of the study participants (n = 240)

Sl. No	Variables		Frequency	Percentage
1	Age group	21-30yrs	21	8.8
		31-40yrs	84	35.0
		41-50yrs	80	33.3
		51-60yrs	49	20.4
		>60yrs	6	2.5
2	Gender	Female	82	34.2
		Male	158	65.8
3	ASA Class	ASA I	61	25.4
		ASA II	179	74.6
4	Comorbidities (Diabetes and Hypertension)	Absent	189	78.8
		Present	51	21.3

Table 2 describes the frequency of study participants according to the modified Brice questionnaire. Amongst them, about 0.8 percent of them conveyed they didn’t fall asleep during the operation. Almost 4/5th of them had a burning or stinging sensation before going to sleep. Only 2.5 percent of patients had a dream during surgery, but no one had a disturbance because of that dream. Nearly 12 percent of them felt that the worst thing about their operation was the recovery process.

**Table 2:** Distribution of study participants according to the modified Brice questionnaire (n = 240)

Sl. No	Variables		Frequency	Percentage
1	Completely asleep for this operation	No	2	0.8
		Yes	238	99.2
2	Last thing the patient remembered before going to sleep	Burning or stinging	194	80.8
		Feeling mask on face	25	10.4
		Smell of gas	21	8.8
3	First thing that the patient remembers after waking up	Feeling breathing tube	210	87.5
		Feeling mask on face	10	4.2
		Feeling pain	15	6.3
		In recovery room	5	2.1
4	Do you remember anything between going to sleep and waking up?	No	235	97.9
		Yes	5	2.
5	Did you dream during the procedure?	No	234	97.5
		Yes	6	2.5
6	Were your dreams disturbing to you?	No	240	100.0
7	What was the worst thing about your operation?	Pain	179	74.6
		Recovery process	29	12.1
		Unable to carryout usual activities	32	13.3

**Figure 1:**
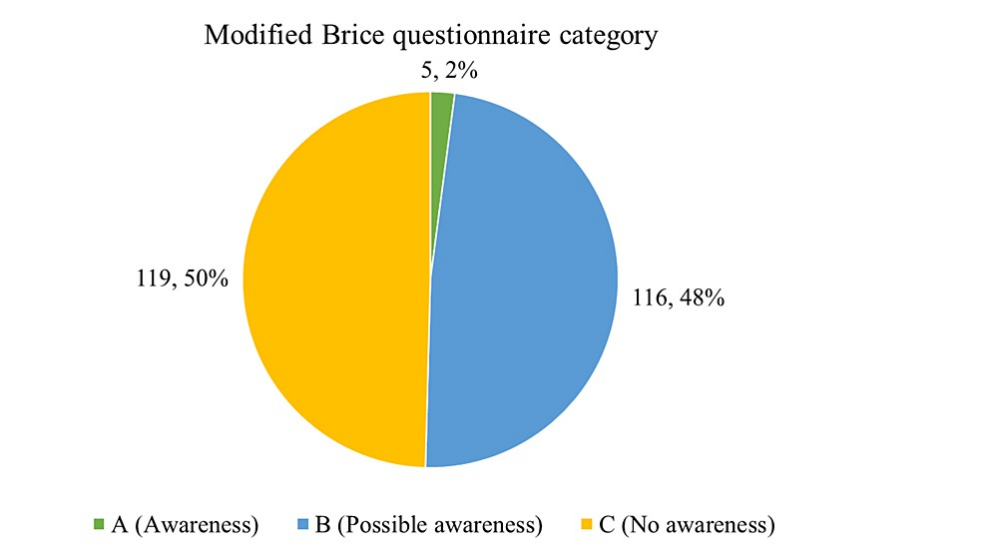
Distribution of the study participants according to the modified Brice questionnaire category (n = 240)

Figure 1 describes the modified Brice questionnaire category among the patients. The incidence of awareness among the study participants was 2 percent.

Table 3 describes the association between the baseline characteristics and the awareness among the study participants. Among the study participants who had comorbidities, 7.8 percent had awareness during surgery when compared to patients who didn’t have comorbidity. This difference in proportion was statistically significant according to the Fischer Exact test (P < 0.0001). There was no association between age group, gender, and ASA status with awareness during surgery in General anesthesia.

**Table 3:** Association between baseline characteristics and Brice's questionnaire category (n = 240)

Sl. No				Brice questionnaire category			Chi-square value	P-Value
				A	B	C		
1	Age group	21 to 30 years	N	1	11	9	6.636	0.559
			%	4.8%	52.4%	42.9%		
		31 to 40 years	N	1	40	43		
			%	1.2%	47.6%	51.2%		
		41 to 50 years	N	1	39	40		
			%	1.3%	48.8%	50.0%		
		51 to 60 years	N	1	24	24		
			%	2.0%	49.0%	49.0%		
		61 to 70 years	N	1	2	3		
			%	16.7%	33.3%	50.0%		
2	Gender	Female	N	2	43	37	1.175	0.564
			%	2.4%	52.4%	45.1%		
		Male	N	3	73	82		
			%	1.9%	46.2%	51.9%		
3	ASA	1	N	0	27	34	2.066	0.323
			%	0.0%	44.3%	55.7%		
		2	N	5	89	85		
			%	2.8%	49.7%	47.5%		
4	Comorbidities	No	N	1	84	104	16.369	< 0.0001
			%	0.5%	44.4%	55.0%		
		Yes	N	4	32	15		
			%	7.8%	62.7%	29.4%		

## Discussion

General anesthesia administered insufficiently to maintain unconsciousness results in awareness. This could happen for several reasons involving surgical and patient considerations, which call for an intentional reduction in anesthetic depth might cause. Pharmacogenetic variables that cause patients to require different doses of anesthesia could also be a contributing factor.

The current study found that about 68 percent of the study participants belonged to the age group of 31 to 50 years, with a predominance of men. The incidence of awareness among the study participants was 2 percent, according to the modified Brice questionnaire. There is an association between awareness during general anesthesia and comorbidities.

In supporting our study results, a review article written by Schwender et al. in 1995 stated that the incidence of awareness among patients undergoing general anesthesia was 0.5 to 2 percent [8].

A study conducted in India in 2017 found results that differed from ours. Singla et al. conducted the study with 896 patients, and they found the incidence to be three in 896 patients, which was 0.33 percent [7]. Another study conducted in India in 2016 by Ambulkar et al. revealed a 0.33 percent incidence of awareness in Maharashtra [9]. This difference might be because of the age difference or difference in the sample size between our study and the above articles. Yet, this discussion needs further research.

In contrast to our study result, a study by Odor et al., in 2021, in the UK, with 3115 patients, found that the incidence was I in 256 patients, which was 0.39 percent. Yet similar tour results conclude that there was an association between comorbidity (low and high BMI) and awareness during surgery under general anesthesia [10]. 

Another cross-sectional study conducted in 2019 in Ethiopia by Tamire et al. with 1065 patients concluded that the incidence of awareness during surgery was 8.2 percent. And they also found that there was no association with the comorbidities. The only association found in that study was between awareness and a lack of pre-medication [11].

In our study, about 2.5 percent of the patients have dreams. In 2021, Parete et al. conducted a study in Karnataka, India, and found that the prevalence of dreams among patients undergoing general anesthesia was 12.5 percent [12]. This difference might be because of the difference in the drug protocol or dosage that is followed in different tertiary care hospitals. Yet, in both studies, the patients claimed the dream had no disturbance. The same study also found that most of the patients claimed pain was the worst thing that happened during surgery [12]. Similar findings emerged from our study, where 75% of patients claimed they had no disturbance in their dreams.

Limitations of the study

Though our study took a fair number of samples, we used convenient sampling in this study. Probability sampling with an estimated sample size yields better results with generalization. In our study, we had not differentiated awareness from hallucination, which can cause bias in the study results. The current study didn't monitor the depth of anesthesia (due to the non-availability of a Bi-spectral monitor [BIS]). We did not consider all the risk factors in this study, which could cause confounding bias and influence the results. Since this study was a cross-sectional study, the association found in this study need not be a causal factor. In our study, for all the study participants, muscle relaxants were administered to the patients; however, Bi-spectral index (BIS) monitoring was not employed to determine the level of anesthesia. Though we used a pre-tested questionnaire for this study, this questionnaire was subjective. There could be a subjective variation, which would influence the incidence got in the current study.

## Conclusions

About two percent of the patients experience awareness of general anesthesia. Nearly three out of 100 patients had experienced dreaming during the procedure. We should take all precautions to avoid becoming conscious while under anesthesia because it is an unpleasant complication that could have long-term psychological repercussions. However, it is good to know that our study results suggest that awareness had an association with comorbidity among the patients undergoing surgery under general anesthesia.

## References

[1] Ghoneim MM, Block RI (1997). Learning and memory during general anesthesia: an update. Anesthesiology.

[2] Sandin RH, Enlund G, Samuelsson P, Lennmarken C (2000). Awareness during anaesthesia: a prospective case study. Lancet.

[3] Sebel PS, Bowdle TA, Ghoneim MM, Rampil IJ, Padilla RE, Gan TJ, Domino KB (2004). The incidence of awareness during anesthesia: a multicenter United States study. Anesth Analg.

[4] Nordström O, Engström AM, Persson S, Sandin R (1997). Incidence of awareness in total i.v. anaesthesia based on propofol, alfentanil and neuromuscular blockade. Acta Anaesthesiol Scand.

[5] Myles PS, Leslie K, McNeil J, Forbes A, Chan MTV (2004). Bispectral index monitoring to prevent awareness during anaesthesia: the B-Aware randomised controlled trial. Lancet.

[6] Samuelsson P, Brudin L, Sandin RH (2007). Late psychological symptoms after awareness among consecutively included surgical patients. Anesthesiology.

[7] Singla D, Mangla M (2017). Incidence of awareness with recall under general anesthesia in rural India: An observational study. Anesth Essays Res.

[8] Schwender D, Klasing S, Daunderer M, Madler C, Pöppel E, Peter K (1995). Awareness during general anesthesia. Definition, incidence, clinical relevance, causes, avoidance and medicolegal aspects. Anaesthesist.

[9] Ambulkar RP, Agarwal V, Ranganathan P, Divatia JV (2016). Awareness during general anesthesia: An Indian viewpoint. J Anaesthesiol Clin Pharmacol.

[10] Odor PM, Bampoe S, Lucas DN, Moonesinghe SR, Andrade J, Pandit JJ (2021). Incidence of accidental awareness during general anaesthesia in obstetrics: a multicentre, prospective cohort study. Anaesthesia.

[11] Tamire T, Demelash H, Yetneberk T, Kibret S (2019). Magnitude and associated factors of awareness with recall under general anesthesia in Amhara Regional State Referral Hospitals, 2018. Anesthesiol Res Pract.

[12] Parate LH, Kaur N, Iyer SS, Geetha CR (2021). The study of postoperative recall in patients under total intravenous anesthesia. Anesth Essays Res.

